# Inflammation-induced reversible switch of the neuron-specific enolase promoter from Purkinje neurons to Bergmann glia

**DOI:** 10.1038/srep27758

**Published:** 2016-06-13

**Authors:** Yusuke Sawada, Ayumu Konno, Jun Nagaoka, Hirokazu Hirai

**Affiliations:** 1 Department of Neurophysiology & Neural Repair, Gunma University Graduate School of Medicine, Maebashi, Gunma 371-8511, Japan; 2 Research Program for Neural Signalling, Division of Endocrinology, Metabolism and Signal Research, Gunma University Initiative for Advanced Research, Maebashi, Gunma 371-8511, Japan

## Abstract

Neuron-specific enolase (NSE) is a glycolytic isoenzyme found in mature neurons and cells of neuronal origin. Injecting adeno-associated virus serotype 9 (AAV9) vectors carrying the NSE promoter into the cerebellar cortex is likely to cause the specific transduction of neuronal cells, such as Purkinje cells (PCs) and interneurons, but not Bergmann glia (BG). However, we found BG-predominant transduction without PC transduction along a traumatic needle tract for viral injection. The enhancement of neuroinflammation by the co-application of lipopolysaccharide (LPS) with AAV9 significantly expanded the BG-predominant area concurrently with the potentiated microglial activation. The BG-predominant transduction was gradually replaced by the PC-predominant transduction as the neuroinflammation dissipated. Experiments using glioma cell cultures revealed significant activation of the NSE promoter due to glucose deprivation, suggesting that intracellularly stored glycogen is metabolized through the glycolytic pathway for energy. Activation of the glycolytic enzyme promoter in BG concurrently with inactivation in PC may have pathophysiological significance for the production of lactate in activated BG and the utilization of lactate, which is provided by the BG-PC lactate shuttle, as a primary energy resource in injured PCs.

Some genes, such as those encoding Neurofilament-M and NeuN (Neuronal Nuclei)^1^, are expressed only in neuronal cells, whereas a different set of genes, such as those encoding glial fibrillary acidic protein and S100β, are expressed specifically in glial cells. Neuron- or glial-cell-specific gene expression is regulated by the respective cell-type-specific promoters. Neuron-specific enolase (NSE), also known as gamma-enolase, is a glycolytic isoenzyme that is found in mature neurons and cells of neuronal origin. The 1.8-kb genomic region upstream of the NSE gene serves as a neuron-specific promoter^2^. Following this initial report, the NSE promoter has been widely used to produce transgenic mice expressing a gene of interest, specifically in neurons^3^,^4^,^5^,^6^,^7^. Moreover, the NSE promoter is used to virally express a transgene specifically in the neurons of mature rodents^8^,^9^,^10^,^11^,^12^.

The cerebellum plays an important role in coordinated movement, motor learning and vestibular function. The cerebellar cortex contains 5 distinct neurons, i.e., granule cells, Purkinje cells (PCs) and three inhibitory interneurons (stellate cells, basket cells and Golgi cells), and is divided into three morphologically distinct parts: the molecular layer, the PC layer, and the granule cell layer (see Fig. 1C,E,H)^13^. The granule cell layer contains numerous granule cells, in which Golgi cells are scattered. The PC layer is a monolayer that consists of the soma of PCs and Bergmann glia (BG), a unipolar astrocyte. The PCs and BG extend their well-differentiated dendrites and processes, respectively, into the molecular layer, where stellate cells and basket cells are located.

When injected into the cerebellar cortex, adeno-associated virus serotype 9 (AAV9) vectors carrying the neuron-specific synapsin I promoter with the minimal CMV promoter specifically transduce cerebellar neurons, such as PCs, stellate cells and basket cells^14^. Thus, the injection of AAV9 vectors incorporating the NSE promoter into the cerebellar cortex is likely to transduce cerebellar neurons but not other cell types, including BG. We verified the neuronal specificity of the NSE promoter by injecting AAV9 vectors carrying the NSE promoter of 1.8 kb in length into the cerebellar cortex. As expected, we detected areas with efficient transduction of PCs and interneurons but not BG. However, to our surprise, there was a distinct region exhibiting the highly efficient transduction of BG concurrent with an almost complete absence of PC transduction, a pattern opposite from the expected neuronal transduction. In this study, we examined the mechanism underlying the reversed transduction pattern driven by the NSE promoter.

## Results

### Astrocyte-predominant transduction by the NSE promoter

**Figure 1:**
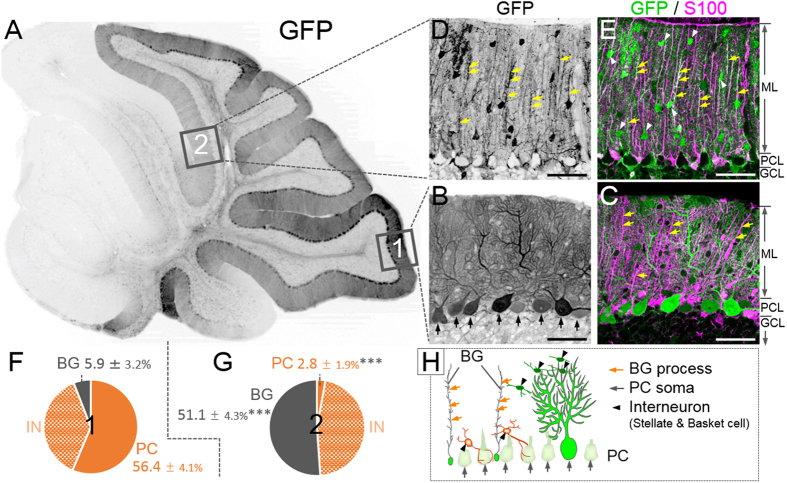
Presence of Bergmann glia (BG)-predominant transduction area in the cerebellum by the neuron-specific enolase (NSE) promoter. Mice received an injection of adeno-associated virus serotype 9 (AAV9) vectors expressing green fluorescent protein (GFP) under the control of the NSE promoter. The cerebellar slices were produced 1 week after viral injection and immunostained for GFP; S100, an astrocyte marker; and parvalbumin, a marker for Purkinje cells (PCs) and interneurons. (**A**) Low-magnified GFP-immunoreactive image of a sagittal section of the cerebellum. (**B–E**) Immunoreactive images for GFP alone (**B,D**) and those for GFP (green) and S100 (magenta) (**C,E**) from region 1 (**B,C**) and region 2 (**D,E**). Note that PCs (arrows in B), but not S100-immunolabelled BGs, in region 1 expressed GFP (**C**), in sharp contrast to no GFP expression in PCs and clear GFP expression in BGs in region 2 (**D,E**). Cells indicated with arrowheads in (**E**) are interneurons. ML, molecular layer; PCL, PC layer; GCL, granule cell layer. (**F,G**) Significant differences were found for the ratio of transduced BG to total transduced cells and for the ratio of transduced PC to total transduced cells between region 1 and region 2. IN; interneuron. Asterisks in (**G**) indicate statistically significant differences compared to region 1 (**F**); ***p < 0.001 via a Tukey post hoc test after a one-way ANOVA. (**H**) An illustration showing the morphology and localization of the cortical cells. Scale bar, 50 μm.

We injected AAV9 vectors expressing GFP under the control of the NSE promoter to the cerebellum of 4- to 5-week-old mice. Mice were euthanised 1 week after viral injection. The sagittal sections were triple immunolabelled for GFP; parvalbumin, a neuronal marker; and S100, an astrocyte marker. As expected in the neuron-specific NSE promoter, fluorescent microscopy of region 1 in the lobule 9 (Fig. 1A–C) revealed efficient and strong GFP expression predominantly in the PCs (arrows, Fig. 1B,H), which were immunolabelled for parvalbumin (data not shown). Surprisingly, we found a negative-positive inverted region (region 2 in Fig. 1A) showing robust GFP fluorescence in S100-labelled BG (yellow arrows indicating BG processes, Fig. 1D,E,H) concomitant with the absence of GFP signal in the PCs. GFP expression in the interneurons, such as stellate cells and basket cells (arrowheads, Fig. 1E,H), was observed in both region 1 and region 2. A quantitative analysis (3 slices/mouse, n = 5 mice) revealed that approximately 94% of all GFP-positive cells in region 1 were neurons, whereas the major GFP-positive cells were BG (51.1 ± 4.3%), with only a small percentage of PCs in region 2 (Fig. 1F,G); the ratio of transduced BG to total transduced cells was significantly higher, and the ratio of transduced PCs to total transduced cells was significantly lower in region 2 than in region 1 (*p* < 0.001). There was no statistically significant difference in the ratios of GFP-expressing interneurons, such as stellate cells and basket cells, to total GFP-positive cells between region 1 (37.7 ± 4.7%) and region 2 (46.0 ± 4.5%).

Almost no S100-positive astrocytes in the granule cell layer expressed GFP in region 1 (PC-predominant transduction area), whereas numerous GFP-expressing S100-positive astrocytes were observed in region 2 (BG-predominant transduction area) (Supplementary Figure 1), suggesting that the NSE promoter is activated not only in BGs but in astrocytes in general.

**Figure 2:**
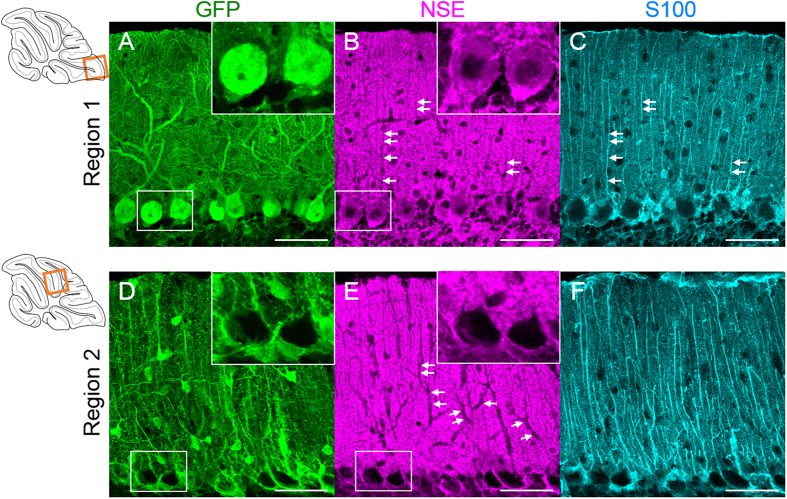
Regulation of the endogenous NSE promoter activity in parallel with that of virally introduced rat NSE promoter. The cerebellar slices treated with AAV vectors expressing GFP under the control of the NSE promoter were immunolabelled for GFP, NSE and S100. (**A–C**) Immunofluorescent images from region 1 (square in an upper left drawing). NSE immunoreactivity was present in the cell bodies of GFP-expressing PCs (insets in **A,B**), whereas it was absent in the BG processes (arrows in **B,C**). (**D–F**) Immunofluorescent images from region 2 (square in a lower left drawing). The NSE immunoreactivity was absent in the cytoplasm (insets in **D,E**) and dendrites (arrows in **E**) of GFP-negative PCs. Scale bar, 50 μm.

The results presented in Fig. 1 clearly indicate that the virally transferred 1.8-kb fragment of the rat NSE promoter is preferentially activated in BG but not in PCs in region 2. Then, we verified whether the endogenous NSE promoter in region 2 was similarly activated in BG and inactivated in PCs. Cerebellar slices from mice 1 week post-viral injection were triple immunostained for GFP, NSE and S100. Confocal laser-scanning images from region 1 showed NSE immunoreactivity in the cytoplasm of the GFP-expressing PCs (Fig. 2A,B, insets) and the absence of NSE immunoreactivity in the BG processes (Fig. 2B,C, arrow). In sharp contrast, NSE immunoreactivity was absent in the somata (Fig. 2E, insets) and dendrites (Fig. 2E, arrow) of PCs lacking GFP expression (Fig. 2D). These results strongly suggest that the endogenous mouse NSE promoter behaves similarly to the virally introduced rat NSE promoter. Because NSE immunoreactivity was detected also in parallel fibres (granule cell axons), it is difficult to distinguish the NSE expression in BG from that in the parallel fibres. Therefore, we used the AAV9 vector-mediated NSE-GFP expression system to further characterize the NSE promoter profile in subsequent experiments.

### A BG-predominant transduction area around lobule 6

**Figure 3:**
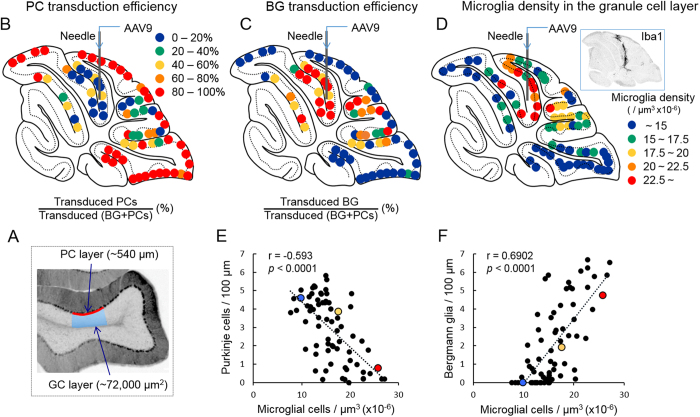
Correlation of the density of transduced PCs or that of BG with adjoining microglial density. (**A**) Schema depicting areas that were used to count PCs, BGs and microglia. The numbers of GFP-expressing PCs and BGs along the approximately 540-μm PC layer (as indicated by a red line) were counted in various transduced areas in the sagittal section of the cerebellum and the number of microglia that were present in the contiguous granule cell layer and half of the medulla (approximately 72,000 μm^2^) just beneath the measured PC layer (as depicted with a blue rectangle). (**B**) Map depicting the PC transduction efficiency. Each dot represents the percent ratio of transduced PCs to transduced (BGs + PCs) in the sagittal section of the cerebellum. Based on the percentage, the ratios were evenly classified into 5 groups with distinct colours as indicated. (**C**) Map depicting the BG transduction efficiency. Each dot represents the percent ratio of transduced BGs to transduced (BGs + PCs) in the sagittal section of the cerebellum. (**D**) Map of the microglial density in different areas of the granule cell layer in the sagittal section of the cerebellum. Based on the cellular density, the results were classified into 5 groups with distinct colours as indicated. Inset shows a representative image of the cerebellar section that was immunolabelled for Iba1, showing strong Iba1 immunoreactivity along the needle track. (**E,F**) Graphs showing the correlation of transduced PC efficiency (**E**) or transduced BG efficiency (**F**) with the density of microglia (Spearman’s rank correlation coefficient, (**E**) r = −0.593, *p* < 0.0001, (**F**) r = 0.6902, *p* < 0.0001). Numbers of GFP-expressing PCs and BG per 100 μm PC layer were plotted against the microglial density in the adjoining granule cell layer (μm^3^ × 10^−6^).

Although the NSE promoter is considered a neuron-specific promoter, one possible explanation for the results above is that the NSE promoter is active in astrocytes and inactive in PCs in a certain region of the cerebellar cortex, including region 2. To examine the transduction profile of the cerebellar cortex by the NSE promoter, we examined the ratios of transduced PCs or transduced BG to transduced [BG + PCs] in different cortical regions of the cerebellum. Approximately 540 μm of the PC layer containing GFP-expressing PCs and BG was randomly selected in a sagittal section of the cerebellum, and the percent ratios of GFP-positive PC or GFP-positive BG to [GFP-positive PCs + GFP-positive BG] in the selected PC layer was determined (Fig. 3A). The results were plotted on drawings of the sagittal sections using the following coloured dots (≧80% = red, ≧60%= orange, ≧40% = yellow, ≧20% = green, and <20% = dark blue) (Fig. 3B,C). The maps revealed that areas in which the NSE promoter was inactive in PC and active in BG were localized around the needle track of the viral injection, suggesting that the switch in the NSE promoter activity from PCs to BG may be triggered by mechanical damage and consequent inflammation.

### Correlation of BG and PC transduction efficiency with microglia density in the contiguous granule cell layer

**Figure 4:**
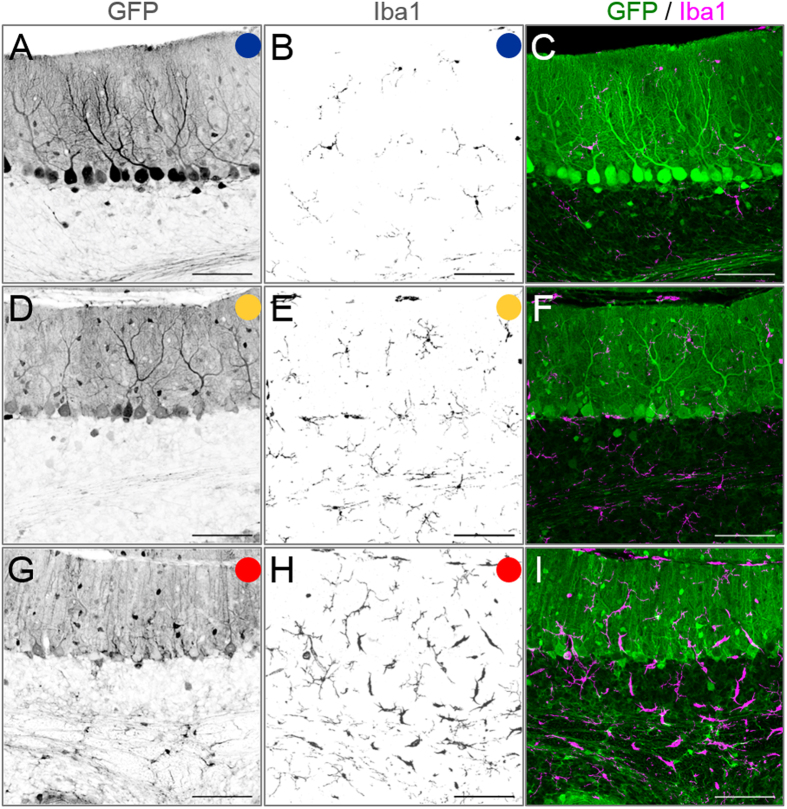
Representative fluorescent images of the cerebellar cortex showing the correlation of the PC or BG transduction efficiency with microglial density. Mice received AAV9 vectors expressing GFP under the control of the NSE promoter. The cerebellar slices that were produced 1 week after the injection were double immunostained for GFP and Iba1, a marker of microglia. Left and middle panels show images that were immunolabelled for GFP and Iba1, respectively. Right panels are merged images of GFP and Iba1. Each image with blue, yellow or red circle corresponds to dots of the same colour as in Fig. 3E,F. Scale bar, 100 μm.

Given that the alteration in the cell-type specificity of the NSE promoter property is caused by mechanical damage and subsequent local inflammation, numerous activated microglia should be observed around BG-dominant transduction areas. To visualize microglia, AAV9 vector-treated cerebellar slices were immunolabelled for Iba1, a marker of microglia. We found a markedly high density of microglia along the needle track inside lobule 6 (inset in Fig. 3D). Then, the microglial density in the transduced areas was measured throughout the sagittal section of the cerebellum: the number of Iba1-immunolabelled microglia in an approximately 72,000 μm^2^ granule cell layer (blue square in Fig. 3A) beneath the PC layer that was used in the analysis of BG/PC transduction efficiency (Fig. 3B,C) was counted. The fluorescent images that were used for this analysis were obtained by the projection of 18 serial sections in 1-μm intervals; thus, the 72,000 μm^2^ area in which the number of microglia was counted had a volume of 1,296,000 μm^3^ (72,000 μm^2^ × 18 μm). Accordingly, the local microglia density was expressed as per [μm^3^ × 10^−6^]. The results were plotted on a drawing of the sagittal section using the following coloured dots (≧22.5 (μm^3^ × 10^−6^) = red, ≧20 = orange, ≧17.5 = yellow, ≧15 = green, and <15% = dark blue) (Fig. 3D). The map showed that high-microglial-density areas almost correspond to BG-predominant transduction areas, suggesting a correlation between the transduction efficiency of BG (or PC) and the microglia density in the adjoining granule cell layer. Then, we plotted the PC density (number of PCs/100 μm PC layer) (Fig. 3E) and the BG density (number of BGs/100 μm PC layer) (Fig. 3F) against the microglia density in the adjoining granule cell layer (μm^3^ × 10^−6^). As expected, the microglia density in the granule cell layer was inversely proportional to the PC transduction efficiency (r = −0.593, *p* < 0.0001) and directly proportional to the BG transduction efficiency (r = 0.6902, *p* < 0.0001) (Fig. 3E,F). Figure 4 shows representative immunofluorescent images for GFP and Iba1 from a PC-predominant transduction area (dark blue), a BG-predominant transduction area (red) and in between (yellow), each corresponding to dots of the same colours as in Fig. 3E,F.

We similarly measured the microglia density in the adjoining PC and molecular layers above the granule cell layer (Supplementary Figure 2). Although activated microglia migrated to the granule cell layer in response to the tissue insult (Fig. 3D), they did not actively enter the molecular layer across the PC layer; thus, there was no statistically significant correlation between the PC or BG transduction efficiency and the microglia density in the contiguous PC and molecular layers (Supplementary Figure 2).

To examine whether the PC to BG switch of the promoter activity due to neuroinflammation was specific to the NSE promoter, we tested the PC-specific mouse L7-4N promoter^15^ and the astrocyte-specific mouse glial fibrillary acidic protein (GFAP) promoter^16^. The AAV9 vectors expressing GFP under either one of the promoters were injected into mouse cerebellum, and the GFP expression profile was examined at 1 week post-injection. Although the needle damage caused microglial invasion, only PCs or BG were transduced by the L7-4N promoter or the GFAP promoter, respectively (Supplementary Figure 3). Thus, the PC to BG switch (or vice versa) was not observed for the L7-4N promoter and GFAP promoter, suggesting that the inflammation-induced switch is specific to the NSE promoter.

### Expansion of the BG-predominant transduction area by enhancing the cerebellar inflammation with LPS

**Figure 5:**
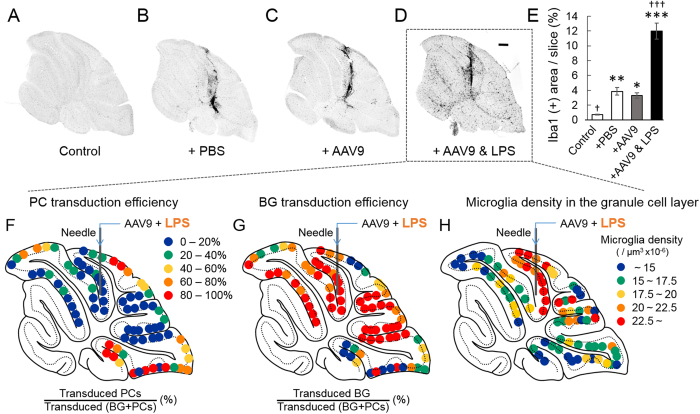
Co-injection of lipopolysaccharide (LPS) with AAV9 vectors augments the local inflammation and expands the areas of BG-predominant transduction and elevated microglia density. Ten microliters of phosphate-buffered saline (PBS) containing LPS (2 μg) and AAV9 vectors or the same volume of AAV9 suspension or PBS alone were injected into the mouse cerebellar cortex; the mice were euthanised 1 week after injection. The cerebellar slices were immunostained for Iba1 to visualize microglia. (**A**) A cerebellar slice from a non-injected control mouse. (**B**) A cerebellar slice from a PBS-injected mouse. (**C**) A cerebellar slice from an AAV9 vector-injected mouse. (**D**) A cerebellar slice from a mouse that was injected with AAV9 suspension containing LPS. Scale bar, 300 μm for (**A–D**). (**E**) The level of inflammation in the slices was assessed by percent ratios of Iba1-immunoreactive area/whole slice area (n = 3 slices/mouse, 3 mice from each experimental group). Asterisks and daggers indicate statistically significant differences compared to a non-injected control and AAV9 vector-injected slices, as determined by a one-way ANOVA followed by a Tukey post hoc test, *p < 0.05, **p < 0.01, ***p < 0.001, ^†^*p* < 0.05 and ^†††^*p* < 0.001. (**F–H**) Map depicting the PC (**F**) and BG (**G**) transduction efficiencies and the microglial density in the granule cell layer (**H**) following application of LPS with AAV9 vectors. Each dot in (**F,G**) represents the percent ratio of transduced PCs (**F**) or BG (**G**) to transduced (BG + PCs) in the sagittal section of the cerebellum.

In addition to inflammation-inducing cytokines and chemokines, tissue damage by needle insertion releases various substances from damaged cells, including kinases, phosphatases and proteases. These substances could influence viral tropism for PCs and BG^17^. Thus, it is still preliminary to conclude that local neuroinflammation and subsequent microglial invasion switches the cell type in which the NSE promoter functions from PCs to BG: inflammation and microglial invasion may be independent of the PC to BG conversion of the NSE promoter activity. To exclude this possibility, cerebellar inflammation was potentiated by injecting AAV9 vectors and lipopolysaccharide (LPS). Initially, we verified whether the co-application of LPS (2 μg) with the viral suspension expanded the size of the inflamed area by examining the degree of microglial activation in the cerebellum. The level of microglial activation was assessed by the percent area that was immunoreactive to Iba1 per total slice area. The percent area that was immunoreactive to Iba1 was significantly larger in PBS-injected slices than in non-injected control slices, but there was no significant difference between the PBS-injected slices and the slices that were injected with PBS containing AAV9 vectors (Fig. 5A–E), indicating that AAV9 vectors do not further augment local inflammation. In contrast, the co-administration of LPS with the viral suspension significantly increased the Iba1-immunoreactive area, indicating the potentiation of cerebellar inflammation by LPS (Fig. 5D,E). Then, we examined BG/PC transduction efficiency by the NSE promoter in the cerebellar sections that were injected with LPS and AAV9 vectors. We found a substantial reduction in the PC-predominant transduction area concomitantly with an expansion of the BG-predominant transduction area (Fig. 5F,G) due to the co-administration of LPS, concurrent with increases in the elevated microglial density area in the granule cell layer (Fig. 5H). These results suggest that local neuroinflammation and subsequent microglial invasion (not limited to a direct traumatic lesion) play key roles in switching the cell type in which the NSE promoter functions from PC to BG.

### Recovery of the neuron specificity of the NSE promoter along with the disappearance of inflammation

**Figure 6:**
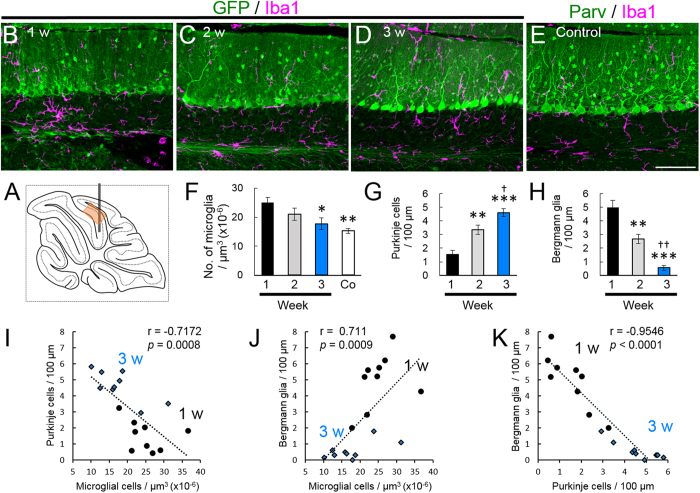
Transduction switch of the NSE promoter from BG to PC, triggered by the disappearance of local inflammation. Mice that received an injection of AAV9 vectors expressing GFP under the control of the NSE promoter to the cerebellum and the sagittal sections were examined via triple immunolabelling for GFP, Iba1 and S100 or parvalbumin 1, 2 and 3 weeks after injection. In addition, non-treated mice were used as controls. (**A**) Schema showing a position that was used for the analysis (an orange square in the lobule 6). (**B–D**) Cerebellar cortical images that were immunostained for GFP (green) and Iba1 (magenta) 1 (**B**), 2 (**C**) and 3 (**D**) weeks after viral injection. (**E**) A cerebellar image that was immunostained for parvalbumin (Parv, green) and Iba1 (magenta) from an age-matched naïve mouse. Scale bar, 100 μm for (**B–E**). (**F**) The number of microglia per μm^3^ × 10^−6^ (microglia density) in the granule cell layer 1, 2 and 3 weeks after viral injection and that from naïve control mice (Co). (**G**) The number of GFP-expressing PCs per 100 μm PC layer 1, 2 and 3 weeks after viral injection. (**H**) The number of GFP-expressing BGs per 100 μm PC layer 1, 2 and 3 weeks after viral injection. (**I,J**) Graphs showing the correlation of transduced PC density (**I**) or transduced BG density (**J**) with the density of microglia (Spearman’s rank correlation coefficient, (**I**) r = −0.7172, *p* = 0.0008, (**J**) r = 0.711, *p* = 0.0009). The numbers of GFP-expressing PCs and BGs per 100 μm PC layer were plotted against the microglia density in the contiguous granule cell layer (μm^3^ × 10^−6^). (**K**) A graph showing the correlation of transduced PC density with transduced BG density (Spearman’s rank correlation coefficient, r = −0.9546, *p* < 0.0001). The circles and diamonds are results that were obtained from the mice at 1 (circles) and 3 (diamonds) weeks after viral injection. Three sections per mouse from 3 mice (total 9 sections) were analysed each week. Asterisks and daggers indicate statistically significant differences compared to the mice at 1 week or 2 weeks post injection, respectively, as determined via a one-way ANOVA followed by Tukey post hoc test, *p < 0.05, **p < 0.01, ***p < 0.001, ^†^*p* < 0.05, ^††^*p* < 0.01.

It would be intriguing to examine whether the neuron specificity of the NSE promoter is restored in parallel with the disappearance of neuroinflammation. To assess the degree of local neuroinflammation, we explored the time course of the microglia density of the granule cell layer in the lobule 6 (Fig. 6A), which experienced substantial mechanical damage and consequent neuroinflammation. We observed numerous activated microglia in accordance with BG-predominant transduction at 1 week after injection (Fig. 6B). The microglia density gradually decreased and became statistically significant 3 weeks after injection (Fig. 6C–F), in parallel with a significant increase in the PC transduction efficiency (Fig. 6G) and a significant decrease in the BG transduction efficiency (Fig. 6H). The microglia density in the granule cell layer 1 and 3 weeks after viral injection was inversely proportional to the PC transduction efficiency (r = −0.7172, *p* = 0.0008) (Fig. 6I) and directly proportional to the BG transduction efficiency (r = 0.711, *p* = 0.0009) (Fig. 6J). In addition, the transduction efficiency of the BG was significantly and inversely correlated with that of the PCs (r = −0.9546, *p* < 0.0001) (Fig. 6K). These results suggest that the degree of local neuroinflammation, i.e., the density of activated microglia, play a key role in determining the NSE promoter activity in PCs and BGs.

### Activation of the NSE promoter by glucose deprivation in cultured C6 glioma cells

**Figure 7:**
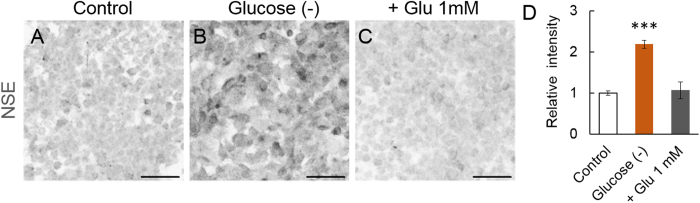
Upregulation of the NSE production by glucose deprivation in C6 glioma cells. The cells were immunostained for NSE at 3 days after the incubation in the medium, as indicated. (**A**) An immunofluorescent image of the cells cultured in the control medium. The cells show weak labelling for NSE. (**B,C**) Immunofluorescent images of glioma cells cultivated in the medium deprived of glucose (Glucose (−)) (**B**) or in the medium containing glucose and 1 mM glutamate (+Glu 1 mM) (**C**). (**D**) Graph showing the relative immunofluorescence intensity of the NSE in glioma cells cultured with indicated conditions. The fluorescence intensity relative to that of control cells was expressed. ***p < 0.001 according to a one-way ANOVA followed by a Tukey post hoc test. Scale bar, 50 μm.

The mechanism by which the NSE promoter is activated in astrocytes was examined using rat C6 glioma cell cultures. The cells were exposed to glucose deprivation or high glutamate (1 mM) to mimic brain injury. Three days after the incubation, the cells were immunostained for NSE. Control C6 glioma cells were weakly immunolabelled for NSE (Fig. 7A), suggesting a slight activity of the NSE promoter in the tumour cells. The glioma cells cultured without glucose showed markedly higher immunofluorescence compared to the control cells (Fig. 7B), whereas the immunofluorescence intensity of the cells cultured in the presence of 1 mM glutamate (and glucose) was comparable to that of the control cells (Fig. 7C). A quantitative analysis of the immunofluorescence intensity revealed that glucose deprivation, but not exposure to high glutamate concentrations, significantly increased the expression levels of NSE (*p* < 0.001) (Fig. 7D). These results imply that limited glucose availability induces NSE production in glioma cells to utilize stored glycogen for an energy resource through the glycolytic pathway.

## Discussion

Currently, several neuron specific promoters are used to generate neuron-specific transgenic mice or for viral vector-mediated transgene expression specifically in neurons, including the synapsin I promoter^11^,^18^,^19^, CaMKII promoter^20^,^21^, tyrosine hydroxylase promoter^22^,^23^ and Thy1 promoter^24^. Although the NSE promoter is widely accepted as a neuron-specific promoter^4^,^25^,^26^,^27^, we showed in this study that the NSE promoter was activated in astrocytes, including BG, but not PC, in the traumatic region of the cerebellar cortex. This PC to BG switch was associated with local microglial invasion; the BG-predominant area was expanded by the LPS-induced potentiation of the inflammation and restored to the inherent PC-predominant transduction as the local inflammation was dissipated.

Then, how does local inflammation trigger the PC to BG switch of the NSE promoter? Traumatic brain injury (TBI) stimulates resident microglia to their activated form^28^,^29^. Activated microglia undergo rapid proliferation and release pro-inflammatory signalling molecules, such as interleukin-1β (IL-1β), tumour necrosis factor α (TNFα), lymphotoxin, matrix metalloproteinases (MMPs), nitric oxide (NO) and other reactive oxygen species^30^, leading to astrocyte activation. TBI also causes the release of glutamate from damaged cells, which over-excites neurons to release further glutamate. The excess amount of glutamate in the extracellular space is primarily taken up by neighbouring activated astrocytes: glutamate is cotransported with Na^+^, leading to the increase in intracellular Na^+^ concentration and the subsequent activation of Na^+^/K^+^ ATPase in astrocytes^31^. To fuel the pump, glycolysis is facilitated in astrocytes, resulting in lactate production, with the subsequent transfer of lactate to neurons (astrocyte-neuron lactate shuttle)^32^. Lactate inhibits glycolysis in neurons through hydroxycarboxylic acid receptor 1 (HCA1), facilitating lactate metabolization into pyruvate^33^,^34^. Thus, TBI causes a critical decrease in the availability of glucose in parallel with the utilization of lactate as an alternative energy resource in neurons. Although glucose has long been considered the only substrate available in brain cells, lactate becomes an alternative substrate to glucose^31^,^35^.

**Figure 8:**
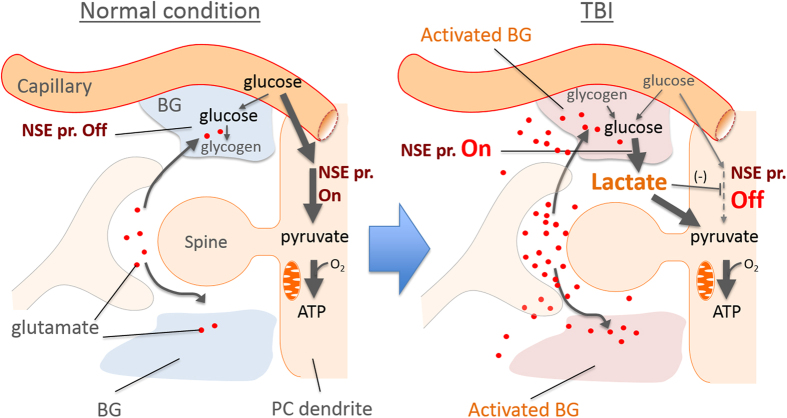
Schematic depicting a possible mechanism regulating the epigenetic switch of the NSE promoter in BG and PCs. In normal conditions, PCs use glucose as a primary energy resource, which is metabolized to pyruvate via the glycolysis pathway, which involves the glycolytic enzyme NSE (NSE promoter is ON). The pyruvate is oxidized and enters the tricarboxylic (TCA) cycle in the mitochondria to generate ATP. In the traumatic brain injury, high amounts of glutamate are released from injured cells, which in turn excites neurons to further release the glutamate. The excess amount of extracellular glutamate is taken up by BG that were activated by cytokines released from activated microglia. In the activated BG, the NSE promoter is activated to produce the NSE, which converts glucose into lactate, with the subsequent transfer of lactate to neurons (astrocyte-neuron lactate shuttle). The lactate inhibits glycolysis in the PCs through hydroxycarboxylic acid receptor 1 (HCA1), leading to suppression of the NSE promoter activity in PCs and facilitating lactate metabolization into pyruvate.

Here, we found that the NSE promoter activity in cultured C6 glioma cells was markedly increased in response to glucose deprivation (Fig. 7). Notably, TBI causes a similar energy dysfunction through a dramatic increase in energy needs and insufficient blood flow. Thus, activation of the NSE promoter in BG around the needle insertion may be explained by a similar epigenetic switch observed in C6 glioma cells. In such a condition, the lactate produced in BG could be transferred to PCs and become a primary energy resource alternative to glucose, which could account for the inactivation of the NSE promoter in PCs (Fig. 8). Consistent with this idea, previous immunohistochemical studies have shown the expression of NSE in reactive astrocytes after brain injury^36^,^37^. Moreover, although it is a peripheral neuron, axonal injury to a hypoglossal nerve in the rat and the cynomolgus monkey decreased NSE immunostaining in the affected neurons 2 to 10 days after the axonal injury: the NSE staining was weakest 10 days after the injury and then began to be restored 20 days after the nerve damage, returning to normal levels by the 45th day^38^.

NSE in both the serum and the cerebrospinal fluid (CSF) can serve as a biomarker for various types of brain injury because NSE is considered to be released from damaged neurons into the CSF and blood in response to brain insult^39^,^40^,^41^,^42^. Indeed, a previous study using a large animal model (swine) of TBI showed that the CSF concentration of NSE increased significantly 6 h after injury, followed by a transient decrease at 24 h and then a second increase for up to 2 weeks after injury^43^. The first increase in NSE in the CSF is likely due to the release from damaged neurons (not from astrocytes). However, given that NSE is synthesized in activated astrocytes concomitantly with the suppression of NSE in certain types of neurons, such as PCs and a hypoglossal nerve^38^, NSE released from activated astrocytes may contribute to the second increase in NSE in the CSF and likely in the serum. This idea is supported by a result in which the concentration of CSF-contained GFAP, an astrocyte-specific protein that is upregulated in activated astrocytes^30^, behaves similarly to NSE in the serum after injury, first increasing at 6 h post-injury, followed by a sustained increase for up to 2 weeks after injury^43^. Although the significance of serum- and CSF-NSE as an indicator of ongoing brain damage is not compromised, the present results suggest the cellular origin of at least a portion of NSE in the serum and CSF as being from activated astrocytes.

## Methods

### Animals

Wild-type C57BL/6 mice between 4 and 5 weeks of age were used in this study. All of the procedures for the care and treatment of the animals were performed according to the Japanese Act on the Welfare and Management of Animals and Guidelines for Proper Conduct of Animal Experiments as issued by the Science Council of Japan. The experimental protocol was approved by the Institutional Committee of Gunma University (No. 09–062). All efforts were made to minimize suffering and to reduce the number of animals that were used.

### AAV9 vector

The region 1.8 kb upstream of the NSE promoter corresponding to a previous report^44^ was cloned from rat genomic DNA (a Wistar rat was kindly provided by Dr. Noriaki Shimokawa). The NSE promoter that was inserted into the AAV expression vector contained the woodchuck hepatitis post-transcriptional regulatory element (WPRE) sequence following GFP to enhance gene expression. For PC- or BG (astrocyte)-specific expression, the NSE promoter in the AAV expression vector was replaced with the 1.0-kb PC-specific mouse L7-4N promoter^15^ or the mouse glial fibrillary acidic protein (GFAP) promoter extending 0.6 kb upstream of the RNA start site (bp + 1)^16^. The AAV9 vector was produced by the co-transfection of HEK293T cells with 3 plasmids, as previously described^14^. The viral particles were purified using ammonium sulphate precipitation and iodixanol continuous gradient centrifugation, as previously described^45^. The genomic titre of the purified AAV9 vector was determined via real-time quantitative PCR using the THUNDERBIRD SYBR qPCR Mix (Toyobo, Osaka, Japan) with the following primers: 5′-CTGTTGGGCACTGACAATTC-3′ and 5′-GAAGGGACGTAGCAGAAGGA-3′ targeting the WPRE sequence.

### Cerebellar injection

After deep anaesthesia via an intra-peritoneal injection of ketamine (100 mg/kg body weight: BW) and xylazine (10 mg/kg BW), mice were placed in a stereotactic frame. For injection into the cerebellum, the skin covering the occipital bone was cut, and a burr hole was made 5 mm caudal from the bregma. The tip of a Hamilton syringe (33 gauge) with an attached micropump (UltraMicroPump II; World Precision Instrument (WPI) Sarasota, FL, USA) was inserted 1.8 mm below the pia mater of the cerebellar vermis. Ten microliters of viral solution (titre; 5.0 × 10^12^ vector genomes (vg)/ml) was injected at a rate of 400 nl/min using a microprocessor-based controller (Micro4; WPI). Two micrograms of LPS extracted from *Escherichia coli* O111:B4 and purified via gel filtration chromatography (L3012; Sigma-Aldrich, St. Louis, MO, USA) was dissolved in 10 μl of PBS containing AAV9 vectors (final LPS concentration; 0.2 μg/μl, AAV9 titre; 5.0 × 10^12^ vg/ml). This dosage of LPS was shown to induce the microglial response in the mouse brain upon intracerebroventricular injection^46^.

### Immunohistochemistry

The mice were euthanised 1, 2 or 3 weeks after viral injection. These deeply anesthetized mice were perfused intracardially with 4% paraformaldehyde in 0.1 M PB (pH 7.4). The whole brains were immersed in 4% paraformaldehyde in 0.1 M PB for 8 h and exchanged in 1×PBS. The cerebellum was cut into 50-μm sagittal sections using a microtome (Leica VT1000 S; Leica Microsystems, Wetzlar, Germany). The slices were blocked with PBS containing 5% normal donkey serum, 0.5% Triton X-100, and 0.05% NaN_3_ (blocking solution) for 30 min and then incubated overnight at 4 °C with the following primary antibodies: rat monoclonal anti-GFP (1:1,000; GF090R; Nacalai Tesque, Kyoto, Japan), mouse monoclonal anti-S100 (1:1,000; S2532; Sigma-Aldrich), sheep polyclonal anti-human and mouse enolase 2 (neuron-specific enolase) (1:500; AF5169; R&D Systems, Minneapolis, MN, USA) and goat polyclonal anti-parvalbumin (1:200; PV-Go-Af460; Frontier Institute, Hokkaido, Japan) or rabbit polyclonal anti-Iba1 (1:1,000; 019–19741; Wako, Osaka, Japan). After washing two and three times with 0.5% and 0.1% Triton X-100 in PBS, respectively, at room temperature, the slices were incubated in blocking solution overnight at 4 °C with the following secondary antibodies (Thermo Fisher Scientific, Waltham, MA, USA): in case of triple immunostaining for GFP, NSE and S100, Alexa Fluor 488 donkey anti-rat IgG (1:1,000), Alexa Fluor 568 donkey anti-sheep IgG (1:1,000) and Alexa Fluor 680 donkey anti-mouse IgG (1:1,000); in case of triple immunostaining for GFP, S100 and parvalbumin, Alexa Fluor 488 donkey anti-rat IgG (1:1,000), Alexa Fluor 568 donkey anti-mouse IgG (1:1,000) and Alexa Fluor 680 donkey anti-goat IgG (1:1,000); and in case of triple immunostaining for GFP, Iba1 and S100, Alexa Fluor 488 donkey anti-rat IgG (1:1,000), Alexa Fluor 568 donkey anti-rabbit IgG (1:1,000) and Alexa Fluor 680 donkey anti-mouse IgG (1:1,000); in case of triple immunostaining for GFP, Iba1 and parvalbumin, Alexa Fluor 488 donkey anti-rat IgG (1:1,000), Alexa Fluor 568 donkey anti-rabbit IgG (1:1,000) and Alexa Fluor 680 donkey anti-goat IgG (1:1,000). After washing two, three and three times with 0.5%, 0.1% Triton X-100 in PBS and 1×PBS, respectively, at room temperature, the immunostained sections were mounted in ProLong Diamond Antifade Mountant with DAPI (P36962, Thermo Fisher Scientific).

### Acquisition of fluorescent images

Fluorescent images were obtained using a fluorescence microscope (BZ-X710; Keyence, Osaka, Japan) or a confocal laser-scanning microscope (LSM 880; Carl Zeiss, Oberkochen, Germany).

### Assessment of transduced PCs, BG and interneurons and microglia

To examine the ratios of transduced PCs or BG to the total transduced cells in PC-dominant and BG-dominant transduction areas (Fig. 1), triple-immunolabelled images for GFP, S100 and parvalbumin were obtained from lobule 9 (PC-dominant transduction area) and lobule 6 (BG-dominant transduction area) using a confocal laser-scanning microscope. Eight sections (45,177.5 μm^2^) at 1-μm intervals were acquired using a 40×objective and Z-stacked by the maximum intensity projection mode of an image-analysing software (ZEN Lite 2012). The numbers of GFP-expressing PCs, BG and interneurons in each image were counted. PCs and interneurons were identified by their morphology and confirmed by their immunolabelling for parvalbumin, whereas BGs were identified by their morphology and confirmed by their immunolabelling for S100. Three slices per mouse and 5 mice (total 15 slices) were analysed. There were no significant differences in the average length of the PC layer that was examined between lobule 9 and lobule 6 (length/area 217.3 ± 0.6 μm in lobule 9 and 216.9 ± 0.7 μm in lobule 6, *p* = 0.69). To examine other regions of the cerebellar cortex (Figs 3, 5, 6 and Supplementary Figure 2), eighteen sections (393,876 μm2) at 1-μm intervals were acquired using a fluorescence microscope (BZ-X710; Keyence, Osaka, Japan) at 20×objective and Z-stacked via image-analysing software (BZ-X analyser; Keyence). The numbers of GFP-expressing PCs, BGs and microglia in each image were counted. PCs were identified by their morphology, having large cell bodies in the PC layer and well-differentiated dendrites extending to the molecular layer, whereas BGs were identified by their morphology and confirmed by immunolabelling for S100. Microglia were identified by immunolabelling for Iba1. Three slices per mouse from 3 mice (total 9 slices) were analysed for Fig. 6, and one slice per mouse from 3 mice (total 3 slices) were analysed for Figs 3 and 5F–H and Supplementary Figure 2. In addition, the length of the PC layer in each image was measured and used to determine the number of PCs or BGs per 100 μm PC layer. The numbers of microglia were counted in the contiguous granule cell layer (approximately 72,000 μm^2^) and in the PC and molecular layers (approximately 100,000 μm^2^). The examined area was 18 μm in depth and thus had a volume. The microglia density in the examined area was expressed as μm^3^ × 10^−6^.

### Percent area occupied by Iba1-immunoreactive microglia in the cerebellar section

Immunofluorescence images of cerebellar slices that were immunolabelled for Iba1 (Fig. 5A–E) were captured using a fluorescence microscope (BZ-X710; Keyence). After parts of the sagittal section were separately acquired at a 10× objective and a 1/12-s exposure time, the pieces were joined together to obtain the stitching image using image-analysing software (BZ-X Analyzer; Keyence). An outline of the whole slice and areas that were immunoreactive to Iba1 was obtained by manually setting an appropriate threshold, and the whole slice and Iba1 immunoreactive areas were measured using ImageJ software. The Iba1-positive area per whole slice area was determined by dividing the former by the latter.

### C6 glioma cells and measurement of the fluorescence intensity

Rat C6 glioma cells^47^ were cultured in Dulbecco’s modified Eagle’s medium (High Glucose) (D-MEM, 044–29765, Wako, Tokyo, Japan) supplemented with 10% foetal bovine serum (172012; Sigma-Aldrich) and 1% penicillin/streptomycin (168–23191; Wako) and maintained in 5% CO_2_ at 37 °C. The cells were plated at a density of 1.0 × 10^5^ cells per well on a coverslip sheet (Cell Desk LF, MS-92132; Sumitomo Bakelite, Tokyo, Japan) for 2 days. For glucose deprivation, the cells were incubated in D-MEM (no glucose) (042–32255, Wako) supplemented with 10% fetal bovine serum and 1% penicillin/streptomycin for 3 days. The cells were immunostained for NSE with essentially a similar protocol as used for cerebellar slices. The NSE immunofluorescence images were captured using a confocal microscope and the same settings from 10 randomly selected regions from 2 independent cultures (5 regions from one culture) in each experimental group. The fluorescence intensity was measured using ImageJ. The background intensity was subtracted from the fluorescence intensity. The averaged fluorescence intensity of control cells was taken as 100%, and the relative values were calculated.

### Statistics

Significant differences were analysed via a Tukey post hoc test after a one-way analysis of variance (ANOVA) using the R software statistical package (www.r-project.org) or Spearman’s rank correlation coefficient using GraphPad Prism 6 (GraphPad Software, San Diego, CA, USA). The data are expressed as the mean ± SEM.

## Additional Information

**How to cite this article**: Sawada, Y. *et al*. Inflammation-induced reversible switch of the neuron-specific enolase promoter from Purkinje neurons to Bergmann glia. *Sci. Rep*. **6**, 27758; doi: 10.1038/srep27758 (2016).

## Supplementary Material

Supplementary Information
