# Supplementary material for: Inflammation-induced reversible switch of the neuron-specific enolase promoter from Purkinje neurons to Bergmann glia
Source: Sci Rep. 2016 Jun 13;6:27758. doi: 10.1038/srep27758 (PMC4904196; doi:10.1038/srep27758)
Supplement: Supplementary Information [file srep27758-s1.pdf]

# Inflammation-induced reversible switch of the neuron-specific enolase promoter from Purkinje neurons to Bergmann glia

Yusuke Sawada[1], Ayumu Konno[1], Jun Nagaoka[1], and Hirokazu Hirai[1,2,*]

[1]Department of Neurophysiology & Neural Repair, Gunma University Graduate School of Medicine, Maebashi, Gunma 371-8511, Japan
[2]Research Program for Neural Signalling, Division of Endocrinology, Metabolism and Signal Research, Gunma University Initiative for Advanced Research, Maebashi, Gunma 371-8511, Japan

***Address correspondence to:** Hirokazu Hirai, Department of Neurophysiology and Neural Repair, Gunma University Graduate School of Medicine, Maebashi, Gunma, 371-8511, Japan
Phone: +81-27-220-7930, Fax: +81-27-220-7936. E-mail: hirai@ gunma-u.ac.jp

**Supplementary Figure 1**

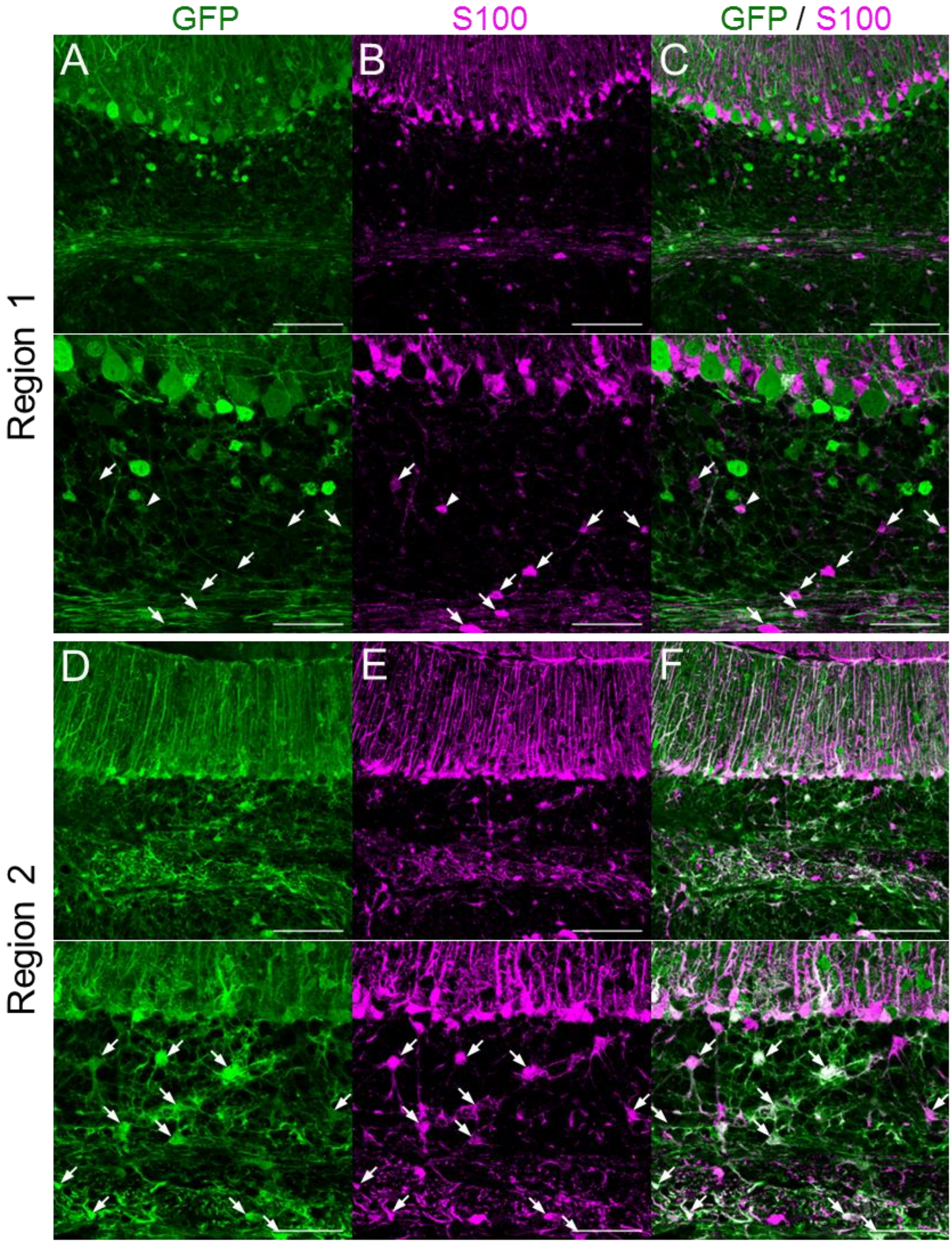


**Supplementary Figure 1**

Activation of the neuron-specific enolase (NSE) promoter in astrocytes present in the granule cell layer and Bergmann glia (BG). Cerebellar sections virally expressing GFP under the control of NSE promoter were double immunolabelled for GFP and S100. (A-

C) Immunofluorescence images obtained from region 1 showing Purkinje cell (PC)-predominant transduction (see Fig. 1A). (D-F) Immunofluorescence images obtained from region 2 showing BG-predominant transduction. S100-positive astrocytes were green fluorescence protein (GFP)-negative in region 1 (arrows in A – C) except for one astrocyte with only faint GFP expression (arrowhead), whereas S100-positive astrocytes in region 2 robustly expressed GFP (arrows in D – E). Scale bars, 100 μm and 50 μm for upper and lower panels, respectively.

## Supplementary Figure 2

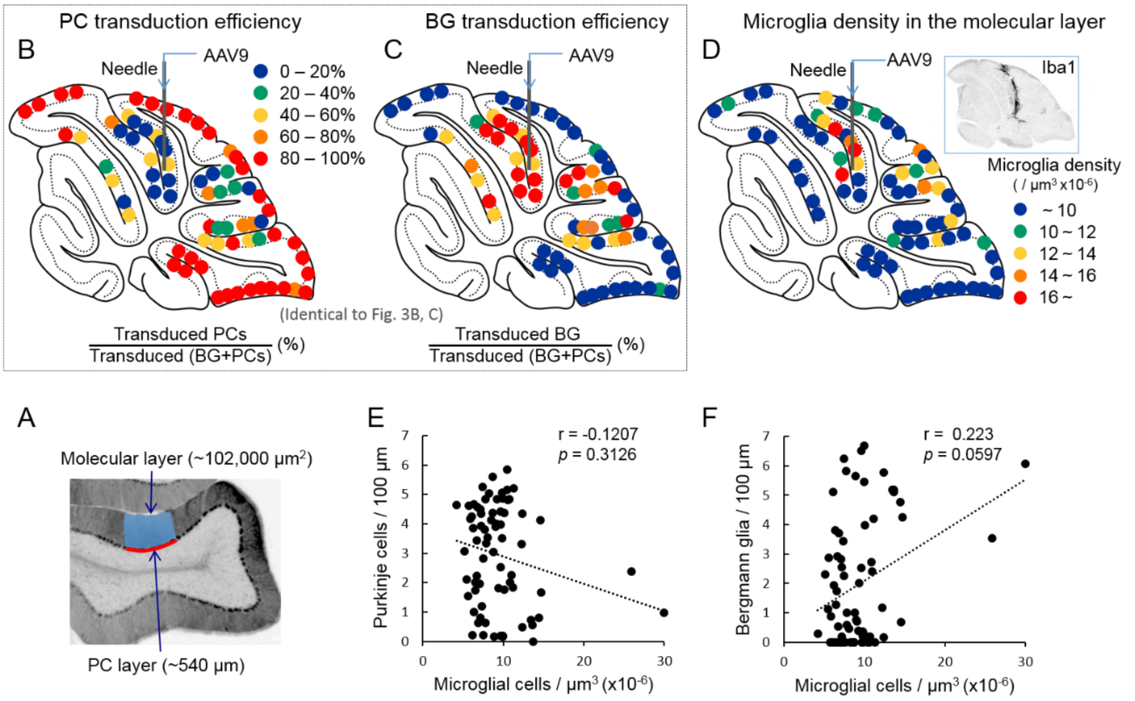


**Supplementary Figure 2**

Absence of correlation of the PC or BG transduction efficiencies with microglia density in the PC layer and in the contiguous molecular layer. (A) Schematic depicting the areas used for counting PCs, BG and microglia. Numbers of green fluorescence protein (GFP)-expressing PCs and BG along the approximately 540-μm PC layer (denoted by the red line) were counted in various transduced areas on the sagittal section of the cerebellum and number of microglia involved in the contiguous PC and molecular layers (approximately 102,000 $\mu m^2$) (denoted by the blue rectangle). (B) Map depicting the PC transduction efficiency (identical to Fig. 3B). Each dot represents the percent ratio of transduced PCs to transduced (BG + PCs) on the sagittal section of the cerebellum. Based on the percentage, the ratios were evenly classified into 5 groups with distinct colours as indicated. (C) Map depicting the BG transduction efficiency (identical to Fig. 3C). Each dot represents the percent ratio of transduced BG to transduced (BG + PCs) on the sagittal section of the cerebellum. (D) Map of the microglia density at different areas of the PC and molecular layers on the sagittal section of the cerebellum. Based on the cellular density, the result was classified into 5 groups with distinct colours, as depicted. Inset shows a representative image of the cerebellar section immunolabelled for Iba1. (E, F)

Graphs showing the correlation of transduced PC density (E) or transduced BG density (F) with the density of microglia. Numbers of GFP-expressing PCs and BG per the 100 μm PC layer were plotted against the microglial density in the contiguous PC and molecular layers ($\mu m^3 \times 10^{-6}$). Note that there was no significant correlation between transduction efficiency of PCs or that of BG and the microglia density in the contiguous PC and molecular layers (Spearman's rank correlation coefficient, (D) r=-0.1207, $p$=0.3126, (E) r=0.223, $p$=0.0597).

**Supplementary Figure 3**

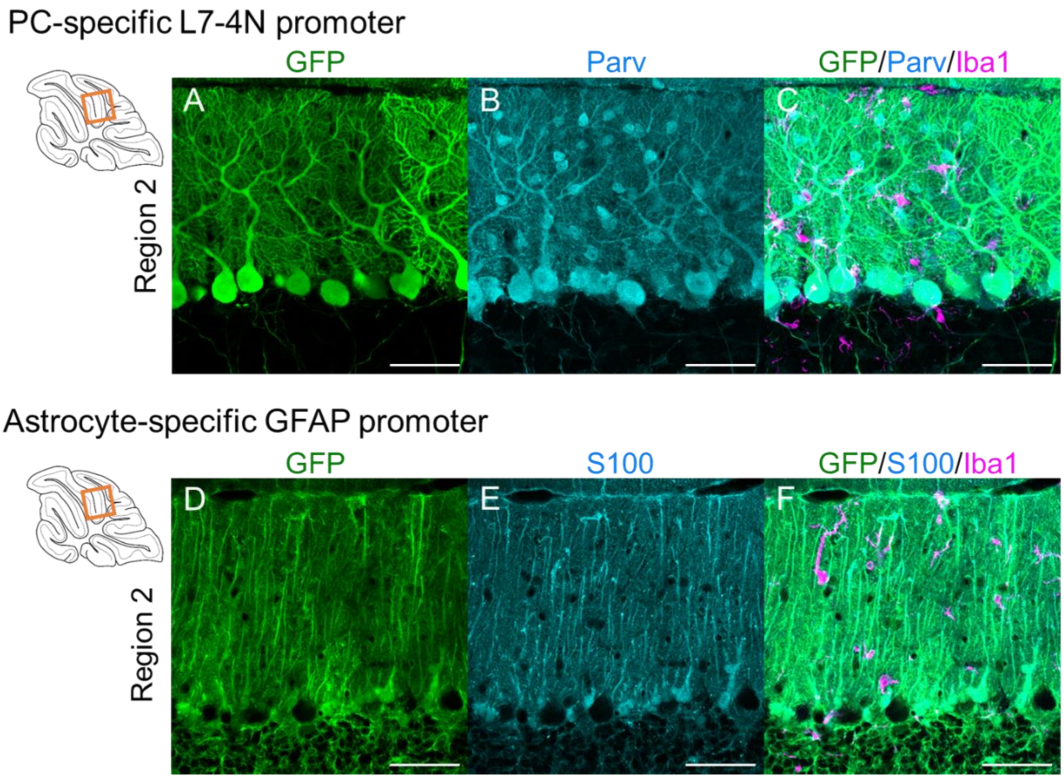


**Supplementary Figure 3**

Absence of PC to BG or BG to PC switch of the PC- and astrocyte-specific promoters in response to the traumatic brain injury. Cerebellar sections were prepared at 1 week after the AAV9 vector injection. (A-C) A cerebellar section from a mouse treated with AAV9 vectors expressing GFP under the control of the PC-specific L7-4N promoter. The sagittal section was triple immunolabelled for GFP, parvalbumin and Iba1. Fluorescent images from region 2 (square in an upper left drawing) are shown. Microglia were infiltrating (C), but GFP expression was observed specifically in the PCs. (D-F) A cerebellar section from a mouse treated with AAV9 vectors expressing GFP under the control of the astrocyte-specific GFAP promoter. The sagittal section was triple immunolabelled for GFP, S100 and Iba1. Fluorescent images from region 2 (square in an upper left drawing) show GFP expression exclusively in the BG despite microglial infiltration (F). Scale bar, 50 μm.
